# Whole-Body Electromyostimulation Improves Performance-Related Parameters in Runners

**DOI:** 10.3389/fphys.2018.01576

**Published:** 2018-11-13

**Authors:** Francisco J. Amaro-Gahete, Alejandro De-la-O, Guillermo Sanchez-Delgado, Lidia Robles-Gonzalez, Lucas Jurado-Fasoli, Jonatan R. Ruiz, Angel Gutierrez

**Affiliations:** ^1^Department of Medical Physiology, School of Medicine, University of Granada, Granada, Spain; ^2^PROmoting FITness and Health through Physical Activity Research Group (PROFITH), Department of Physical Education and Sports, Faculty of Sport Sciences, University of Granada, Granada, Spain

**Keywords:** WB-EMS, VO_2_max, running economy, detraining, recreational runners, endurance

## Abstract

The aim of this study was to study the effects of a 6-session (one per week) WB-EMS training intervention on maximum oxygen uptake, aerobic and gas exchange thresholds, running economy, and muscular power in male recreational runners. Twelve men were randomized into WB-EMS intervention (*n* = 6; 27.0 ± 7.5 years; 70.1 ± 11.1 kg; 1.75 ± 0.5 m) or control (*n* = 6; 27.0 ± 6.1 years; 73.6 ± 3.4 kg; 1.77 ± 0.3 m). The WB-EMS group reduced the running training frequency to one per week and followed one WB-EMS training session per week during 6 weeks. Participants in the control group maintained their usual running endurance training. Each participant completed four assessments: physiological parameters [(i) VO_2_max, aerobic and gas exchange threshold values, and (ii) running economy at two intensities], muscular power (vertical jump), and anthropometric parameters both at baseline and after the intervention. Participants in the WB-EMS group improved VO_2_max, aerobic and gas exchange threshold values, running economy, and vertical jump (*p* < 0.05) compared to the control group. There, WB-EMS seems to be an effective training methodology leading to improvements in performance during endurance training volume reduction in male recreational runners.

## Introduction

Long-distance running performance depends on the interaction of physiological, biomechanical, and psychological factors (Bassett and Howley, 2000). Physiological attributes include (i) high cardiac output and high rate of oxygen availability and delivery to working muscles reflected on maximum oxygen consumption (VO_2_max) and dependent of muscle capillary density, stroke volume, maximal heart rate, and hemoglobin content; (ii) capacity to sustain a high VO_2_ fraction for long periods of time [i.e., ventilatory threshold 1 (VT1) and ventilatory threshold 2 (VT2), which depend on aerobic enzyme activity, and distribution of power output]; (iii) capacity to produce movement with the minimum energy cost [i.e., running economy (RE), which depends on the percentage of slow twitch muscle fibers, anthropometry, and elasticity] (Bassett and Howley, 2000; Foster and Lucia, 2007; Joyner and Coyle, 2008); and (iv) capacity to develop muscular power (Nuhr et al., 2003). Detraining has been characterized as a partial loss of training-induced physiological and performance adaptations, as a consequence of various weeks of training cessation (Maldonado-Martín et al., 2016). However, detraining periods are frequent and, in many cases, uncontrollable in most sport disciplines (injuries, transition periods, discharge micro-cycle, etc.). It has been shown that detraining periods produce several decrements in VO_2_max after 3 and 8 weeks (7% and 16%, respectively) (Costill et al., 1985; Mujika and Padilla, 2001). Therefore, it is necessary to seek for other alternatives to prevent or reduce the large decreases in physiological and performance-related parameters induced by training cessation (Mujika and Padilla, 2001; Berryman et al., 2018).

Strength training has emerged as an effective strategy to improve aerobic running performance (Mujika, 2017; Berryman et al., 2018). Although local electromyostimulation training seems an alternative to traditional strength training (Filipovic et al., 2012), there are no studies that evaluate its effects on physiological parameters related physical fitness in recreational runners.

Whole-body electromyostimulation (WB-EMS) is becoming increasingly popular as a novel training technology. While local electromyostimulation produces an external stimulation of single specific muscle groups, WB-EMS is able to simultaneously stimulate up to 14–18 regions or 8–12 different muscle groups with up to 2.800 cm^2^ electrode area (Filipovic et al., 2015). Positive effects of WB-EMS on health biomarker parameters have been shown. WB-EMS improves body composition in elderly women (≥60 years) with sarcopenic obesity or at risk of sarcopenia (Kemmler et al., 2014, 2016c), in postmenopausal (Kemmler et al., 2010) and in healthy untrained middle-aged men (Kemmler et al., 2016b). Secondly, WB-EMS increases resting metabolic rate in postmenopausal women (Kemmler et al., 2010), and increases energy expenditure during exercise in moderately trained men (Kemmler et al., 2012). WB-EMS also improves strength levels in elite football players (Filipovic et al., 2016), in postmenopausal women (Kemmler et al., 2010), and in healthy untrained middle-aged men (Kemmler et al., 2016b), as well as bone mineral density in osteopenic women (>70 years) (Von Stengel et al., 2015). Finally, WB-EMS training seems to improve human red blood cell deformability in elite football players (Filipovic et al., 2015). Whether WB-EMS is able to improve running performance parameters is unknown, and whether it is able to prevent or reduce performance-related detraining after a period of training volume reduction remains to be investigated. However, considering that previous studies have demonstrated that WB-EMS can enhance muscular strength (Kemmler et al., 2010, 2014; Filipovic et al., 2016) and body composition (Kemmler et al., 2010, 2014, 2016b,c) by an increment of total muscle contraction during a training session, and that the development of muscular strength and body composition are related to endurance performance (Mujika, 2017; Berryman et al., 2018), it seems plausible that a well-designed WB-EMS training program can induce an improvement of endurance performance-related parameters. A possible physiological explanation that support this idea could be that the extra activation produced by WB-EMS may induce better neural function, peripheral changes such as a shift in muscle-fiber distribution (from fast twitch type IIb toward fatigue-resistant type IIa) and increases in muscle–tendon stiffness improving endurance performance.

To note, most of the intervention programs applied in scientific studies using WB-EMS consisted of a set of non-functional exercise tasks (i.e., not considering the motor requirements of specific sport or functional movements). Moreover, these programs did not modify electrical or training parameters across sessions. This lack of stimuli variation could lead to a decrease in training efficiency (Harries et al., 2015), and poor sport performance (Kiely, 2012). Thus, there is a need to study whether a WB-EMS intervention based on functional exercises (considering the motor requirements of specific sport) and with electrical parameters following a periodization improves running performance.

The aim of this study was to determine the effects of a 6 weeks (once session per week) WB-EMS training program on VO_2_max, VT1, VT2, RE, and vertical jump in recreational runners, while participants reduced the running frequency training to once per week. Our hypothesis, is that WB-EMS training program could keep and even improve running performance despite a relative running frequency training reduction.

## Materials and Methods

### Experimental Approach

The present study is part of a parallel randomized controlled trial that followed the CONSORT statements (ClinicalTrials.gov ID: NCT03425981). The first part of this project that studied the effects of a periodized and functional WB-EMS training on VO_2_max, VT1, VT2, RE, and vertical jump in runners compared with a traditional WB-EMS was published recently (Amaro-Gahete et al., 2018). The present study investigated the effects of the periodized and functional WB-EMS training modality on running performance with a control group. Participants in the WB-EMS group were instructed to reduce their running training program volume, whereas the CG continued with their running training in term of volume and intensity: two or three times per week (45–60 min per day) at an intensity of 60–70% heart rate reserve, which was controlled by heart rate monitor (Polar RS300X, POLAR, Kempele, Finland), and with 24–48 h of rest between sessions. Nevertheless, there is some overlap between the two publications with respect to participants and general methodology.

### Participants

Fourteen healthy male recreational runners (26.6 years; BMI = 23.5 kg/m^2^) participated in the study. Participants were frequent runners (running frequency of two to three times per week, at least 90–180 min/week) and none had received WB-EMS training. Two out of fourteen participants did not complete the study and were excluded from the analysis. Participants signed an informed consent to participate in the study and no ethical issues were raised in relation to the type of measurements to be performed in the study. The study was approved by the Human Research Ethics Committee of the University of Granada (200/CEIH/2016) and complied with the ethical guidelines of the Declaration of Helsinki, last modified in 2013. Participants were instructed not to modify their nutrition habits.

### WB-EMS Training Program

The WB-EMS device used (Miha Bodytec, Augsburg, Germany) allows to modify several electrical parameters: (i) frequency, defined as the number of electrical pulses per time unit. Several studies shown that slow and fast fibers are not selectively activated with local electromyostimulation at low or high frequencies, but it is well known that it preferentially recruits fast versus slow motor units independently of the frequency applied (Gregory and Bickel, 2005), (ii) impulse width, which could influence the intensity of muscle contraction when combined with other stimulation parameters in the recommended range (Filipovic et al., 2012; Amaro-Gahete et al., 2017); (iii) impulse intensity, which was adjusted (at the subject’s maximum tolerance levels) because of the increasing tolerability of the current intensity, following the guided and supervised low-intensity resistance protocols recently described (Kemmler et al., 2016a) every 3–5 min in close cooperation with the participants (Kemmler et al., 2010, 2012, 2014, 2016b,c; Kemmler and von Stengel, 2013; Von Stengel et al., 2015); and (iv) duty cycle, which is defined as the ratio between time receiving electrical stimuli and the total cycle time (in relation to the frequency selected, as high duty cycle need to be used with low frequencies to be feasible) (Filipovic et al., 2012; Amaro-Gahete et al., 2017).

The WB-EMS equipment enables the simultaneous activation of sixteen different muscle groups (e.g., upper legs, upper arms, gluteals, abdomen, chest, lower back, upper back, shoulder; total size of electrodes: at least 2,800 cm^2^). The WB-EMS training program consisted of six WB-EMS training sessions (one per week) and six running training sessions (also one per week). All WB-EMS training sessions were short (<20 min) and a high intensity training approach was applied. Participants went through a familiarization session (prior to the exercise program) aiming to learn movement patterns (i.e., proper techniques of the exercises) and adapt to the electric stimuli. Electrical parameters (frequency, impulse intensity, and duty cycle), volume (training session time and work-recovery time), and perceived intensity (RPE) were gradually increased along the 6 weeks of intervention. Despite several reviews have provided information about efficacy ranges of most common electrical parameters in local electromyostimulation (Gregory and Bickel, 2005; Maffiuletti, 2010; Filipovic et al., 2011), the rationale of our periodization was based on the principle of progression, because it is not well known which are the best specific values of each electrical parameter in WB-EMS training for improving running performance. Participants were asked to report the intensity of the electric impulse training and the perceived intensity by using the RPE scale (Borg, 1982).

Running training sessions consisted in 20 min running at two different intensities; 10 min at VT1 speed and 10 min at 90% of VT2 speed, and these running sessions were also performed by the control group as a part of its running training volume per week. All WB-EMS sessions were supervised by an experienced National Strength and Conditioning Association Certified Personal Trainer (NSCA-CPT).

This intervention program followed a within-day undulating periodization model. The training sessions were divided into four parts: warm up (phase A), strength training part (phase B), high intensity interval power training part (phase C), and high intensity interval training part (phase D). In all cases, participants only did movements when receiving electrical impulse. Electrical parameters were modified throughout different parts of the training program and throughout the session (see Table 1). A circuit training methodology was applied (with no rest between exercises) in all phases and no external load was used. In phase A, participants performed 7–10 repetitions (one set) of three exercises (e.g., ½ squat and arm curl); both concentric and eccentric phases lasted 2 s each in every repetition. In phase B, participants performed one to two sets of 5–10 repetitions of six exercises (e.g., Bulgarian squat and military press); concentric phase lasted one second and eccentric phase duration was three seconds. In phase C, participants performed one set of eight exercises (e.g., climber); they were instructed to do as many repetitions as possible in 10 s with a 10-s rest between exercises. In phase D, participants did one to two interval sets running on a treadmill with two different intensities: moderate intensity (65% VO_2_max speed, 30..) and high intensity (>85% VO_2_max speed, 30..). Specific exercises of each training session have been largely described in a previous study (Amaro-Gahete et al., 2018).

**Table 1:** Electric parameters description in WB-EMS sessions (periodization).

WB-EMS/SESSION	1				2				3				4				5				6			
	W	S	HP	HT	W	S	HP	HT	W	S	HP	HT	W	S	HP	HT	W	S	HP	HT	W	S	HP	HT
Total duration (min)	2	6	2	2	4	6	3	3	4	8	3	3	4	8	4	4	4	8	4	4	4	8	4	4
Frequency (Hz)	12	55	60	20	12	65	70	25	12	75	80	35	12	85	90	40	12	85	90	40	12	85	90	40
Impulse width (μs)	350	350	350	350	350	350	350	350	350	350	350	350	350	350	350	350	350	350	350	350	350	350	350	350
RPE impulse intensity (6–20)	10	12	13	13	10	12	13	13	10	14	15	15	10	16	17	17	10	16	17	17	10	16	17	17
Duty cycle (%)	50	50	50	50	50	50	50	50	50	50	50	50	50	50	50	50	50	50	50	50	50	50	50	50
	4:4	4:4	10:10	30:30	4:4	4:4	10:10	30:30	4:4	4:4	10:10	30:30	4:4	4:4	10:10	30:30	4:4	4:4	10:10	30:30	4:4	4:4	10:10	10:10

*WB-EMS, whole-body electromyostimulation training; W, warm-up phase; S, strength phase; HP, high intensity interval power training phase; HT, high intensity interval training phase; RPE, rated perceived exertion; Min, minutes; Hz, hertz; μs, microseconds; mA, milliamps.*

### Assessments of Dependent Variables

**FIGURE 1:**
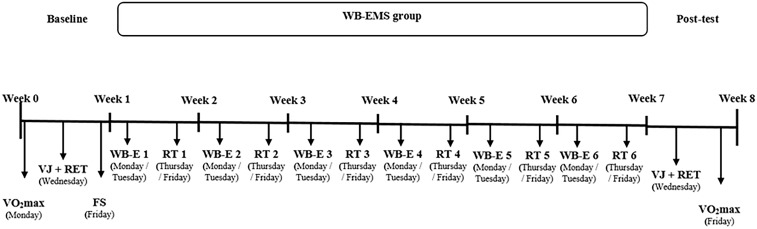
Experimental timeline: Testing period in week 0 and week 7, training and running economy from week 1 to week 6. WB-EMS, whole-body electromyostimulation; VO_2_max, maximal treadmill exercise test; VJ, vertical jump test; RET, running economy test; FS, familiarization session; WB-E, whole-body electromyostimulation training session; RT, running training session.

Before and after the intervention, participants were examined during 2 days. On day 1, an anthropometric assessment and a maximal treadmill exercise test were performed; on day 2, a vertical jump test and a running economy test were conducted (Figure 1). Assessments were performed at the same time of day (midmorning) to avoid diurnal variation in performance and changes in laboratory conditions (19–22°C temperature; 45–55% relative humidity). Subjects were asked to consume their habitual diet and to avoid alcohol, caffeine, and vigorous-intensity exercise in the 48 h prior to assessments days. Day 1 and day 2 were separated by 48 h.

### Anthropometry

Body mass was determined with an accuracy of 100 g on a SECA scale (SECA, Hamburg, Germany) and height was determined with an accuracy of 0.100 cm with a SECA stadiometer (SECA, Hamburg, Germany); and body mass index was calculated (kg/m^2^).

### Maximal Oxygen Consumption

Maximal oxygen consumption was assessed using a maximum treadmill (H/P/Cosmos Pulsar treadmill, H/P/Cosmos Sport and Medical GMBH, Germany) exercise test with a progressive incremental protocol that has been extensively used and validated (Machado et al., 2013). In brief, after a warm-up consisting in walking at 5 km/h for 3 min, protocol started with an initial speed of 8 km/h, which was increased 1 km/h every minute until participants reached their volitional exhaustion. Thereafter, participants underwent a cooling-down period (4 km/h during 5 min). O_2_ uptake and CO_2_ production were measured with a gas analyzer (Oxycon Pro; Jaeger, Höchberg, Germany). The gas analyzer was calibrated with a known gas mixture (0% O_2_ and 5.5% CO_2_) and environmental air (20.9% O_2_ and 0.03% CO_2_) immediately before each test. Participants were strongly encouraged to invest maximum effort consistently across assessments. Participants were previously familiarized with the 6–20 Borg scale (Borg, 1982), which was used to measure the RPE during the last 15 s of each stage and at exhaustion. Heart rate (HR) was recorded every 5 s (Polar RS300, Kempele, Finland) and maximum heart rate (HRmax) was defined as the highest recorded HR value (Tanaka et al., 2001). We also registered respiratory, RPE, and heart rate parameters during cooling-down period. Serum lactate was measured by a Lactate Pro analyzer (Fact Canada, Quesnel, BC, Canada) 3 min after the volitional exhaustion was reached. The electrocardiogram was continuously monitored. Gas exchange data was averaged each 10 s and was downloaded for later analysis. Poole and Jones (2017) recommended to check VO_2_max plateau with a constant work rate test performed at 110% of the work rate achieved after ramp protocol test. However, we decided not to include it because our participant were recreational runners and this validation is important specially in sedentary people or patients (Poole and Jones, 2017).

### Ventilatory Thresholds

VT1 and VT2 were estimated from gas exchange data through different respiratory variables: minute ventilation (VE) and equivalents for oxygen (VE/VO_2_) and carbon dioxide (VE/VCO_2_) by two independent researchers (FAG and AGS). A third researcher opinion was sought when they disagreed (AOP). VT1 was determined at the first point where an increase in VE/VO_2_ with no increase in VE/VCO_2_ and the departure from linearity of VE occurred (Lucía et al., 2000). VT2 was determined at the first point where an increase in both the VE/VO_2_ and VE/VCO_2_ occurred (Lucía et al., 2000). Speed at VT1 and VT2 was determined and VO_2_max percentage in VT1 and VT2 were also calculated.

### Running Economy

Running economy was determined during a treadmill test following a specific protocol used in previous studies (Foster and Lucia, 2007; Shaw et al., 2014). The treadmill test consisted of two 10-min stages at two different intensities. The two stages were performed at the speed where VT1 and the 90% of VT2 were reached in the pre-intervention maximum treadmill exercise test. The first 2 min of each stage were discarded. RE (oxygen cost of running a kilometer at a specific velocity) was calculated using the following equation: VO_2_ (ml/kg/min)/[speed (km/h)/60] (Foster and Lucia, 2007; Shaw et al., 2014).

### Muscular Power

Vertical jump performance was assessed using the countermovement jump (CMJ) and Abalakov jump (ABJ) tests. Jumping height was calculated from flight time using kinematic equations (Lehance et al., 2005) estimated by Ergo Jump Bosco System^®^ (Globus, Treviso, Italy). Before carrying out the tests, a standardized warm-up was performed which included 5 min run at 50% of heart rate reserve, mobility and muscle activation exercises.

To perform the CMJ, participants were instructed to start in a standing position without arm swing; they performed a 2-leg CMJ consisting in a fast-downward movement to a freely chosen angle, immediately followed by a fast-maximal vertical thrust. Any jump that was perceived to deviate from the required instructions was repeated. Two trials separated by 1 min of passive recovery were performed. The highest jump was considered for further analysis. For the ABJ, participants did the same actions than CMJ, but including arm swing.

### Statistical Analyses

All outcome variables were checked for normality with a graphical test (QQ-Plots) and results expressed as mean and SD. Baseline and post intervention data were compared using Student’s paired *t*-test. One-way analysis of covariance (ANCOVA) was used to examine the effect of treatment group (fixed factor) on performance-related parameters. Multiple comparisons were adjusted according to Bonferroni. We conducted all statistical analyses using SPSS Statistics (version 20, IBM, Ehningen, Germany) software, setting level of significance at *p* < 0.05.

## Results

Participants completed 95.83% training sessions in WB-EMS. No WB-EMS related adverse effects were reported by any participant. Baseline characteristics, post-intervention values, and mean change of study performance-related variables and body composition parameters in WB-EMS and control groups are listed in Table 2. There was no difference between groups before the intervention program. The impulse intensity reported by the participants for each session (measured by RPE) was similar to the impulse intensity pre-established (mean difference ± SD, 0.84 ± 0.24). This fact confirmed that participants of WB-EMS group adhered to the protocol designed in term of impulse intensity. Detailed information of the training performed by control group during the intervention study is listed in Table 3.

**Table 2:** Descriptive parameters and changes in primary and secondary outcomes following a 6-week training program.

	WB-EMS (*n* = 6)			CONTROL GROUP (*n* = 6)		
	PRE	POST	*P*-value	PRE	POST	*P*-value
Body mass (kg)	70.1 ± 11.1	68.6 ± 11.0	0.023^∗^	73.6 ± 2.1	74.1 ± 1.9	0.083
BMI (kg/m^2^)	22.6 ± 2.8	22.2 ± 2.6	0.031^∗^	23.4 ± 0.8	23.5 ± 0.7	0.060
VO_2_max (ml/min)	3790.8 ± 812.4	3894.8 ± 802.4	0.082	3905.8 ± 480.2	3905.0 ± 412.6	0.983
VO_2_max (ml/kg/min)	53.9 ± 5.3	56.7 ± 5.2	0.001^∗∗^	53.1 ± 6.4	52.7 ± 5.6	0.442
VO_2_ VT1	28.2 ± 3.4	29.6 ± 3.4	0.010^∗∗^	27.0 ± 3.6	26.8 ± 3.4	0.399
VO_2_ VT2	40.8 ± 4.6	43.7 ± 5.1	0.004^∗∗^	40.6 ± 3.3	40.1 ± 3.7	0.325
%max VO_2_ VT1	56.3 ± 8.5	58.8 ± 12.1	0.336	54.2 ± 5.4	54.9 ± 2.8	0.797
%max VO_2_ VT2	84.2 ± 5.4	87.2 ± 7.1	<0.001^∗∗∗^	85.2 ± 4.5	83.9 ± 3.5	0.101
SPEEDpeak (km/h)	16.7 ± 1.6	17.5 ± 1.8	0.041^∗^	15.5 ± 1.6	15.5 ± 0.8	1.000
VT1s (km/h)	9.3 ± 1.2	10.3 ± 1.4	0.042^∗^	8.3 ± 0.5	8.5 ± 0.5	0.611
VT2s (km/h)	14.0 ± 1.1	15.2 ± 0.8	0.001^∗∗^	13.2 ± 1.2	13.2 ± 1.2	0.511
RE VT1 (ml/kg/km)	210.1 ± 12.0	202.4 ± 12.5	0.001^∗∗^	220.5 ± 20.5	219.8 ± 19.6	0.465
RE VT2 (ml/kg/km)	248.5 ± 26.3	233.1 ± 22.6	0.001^∗∗^	258.7 ± 21.2	260.1 ± 23.7	0.340
CMJ (m)	0.32 ± 0.06	0.33 ± 0.06	0.027^∗^	0.32 ± 0.04	0.32 ± 0.03	0.967
ABJ (m)	0.36 ± 0.06	0.38 ± 0.06	0.001^∗∗^	0.37 ± 0.01	0.37 ± 0.01	0.789

*^∗^P < 0.05, ^∗∗^P < 0.01, ^∗∗∗^P < 0.001, Student’s paired t-test. BMI, body mass index; VO_2_max, maximum oxygen uptake; %max VO_2_ VT1, oxygen uptake percentage in ventilatory threshold 1 relative of maximum oxygen uptake; %max VO_2_ VT2, oxygen uptake percentage in ventilatory threshold 2 relative of maximum oxygen uptake; VT1s, ventilatory threshold 1 speed; VT2s, ventilatory threshold 2 speed; CMJ, countermovement jump; ABJ, Abalakov jump; RE VT1, running economy ventilatory threshold 1; RE VT2, running economy at 90% of ventilatory threshold 2; WB-EMS, whole-body electromyostimulation group.*

**Table 3:** Detailed information of the training performed by control group during the intervention study.

	Training session (session/weeks)	Session duration (min)	Session intensity (%HRres)
Week 1	2.7 ± 0.3	51.7 ± 6.2	64.8 ± 3.1
Week 2	2.6 ± 0.4	48.3 ± 8.0	61.4 ± 7.8
Week 3	2.7 ± 0.5	53.1 ± 4.6	62.8 ± 4.7
Week 4	2.5 ± 0.4	49.5 ± 5.2	66.7 ± 5.7
Week 5	2.8 ± 0.3	50.3 ± 7.3	67.1 ± 3.8
Week 6	2.4 ± 0.5	54.4 ± 5.7	65.7 ± 4.3

*Results are expressed as mean ± SD. HRres, heart rate reserve.*

**FIGURE 2:**
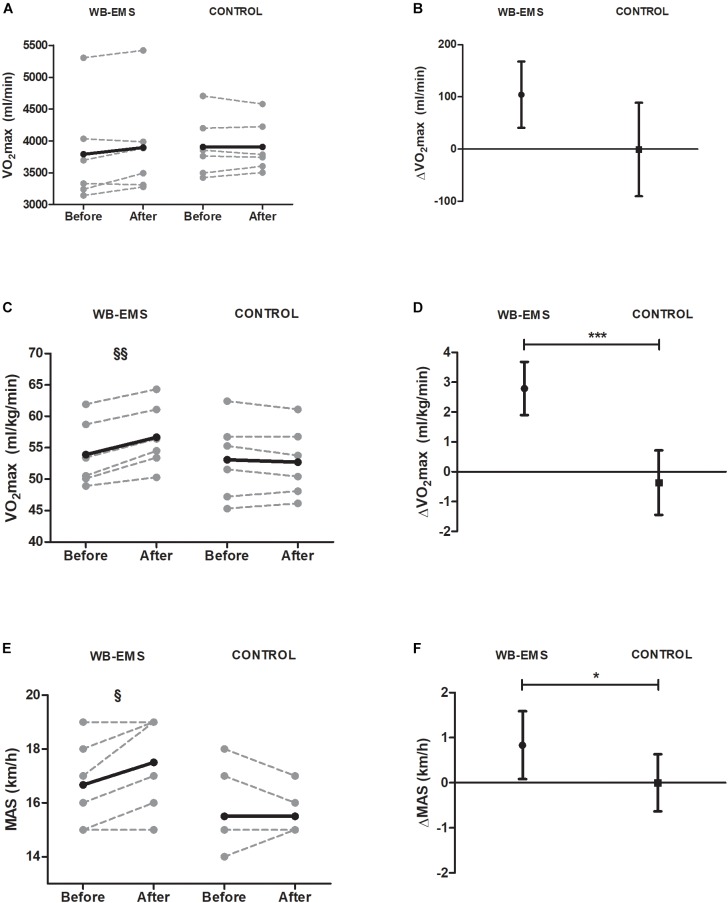
Pre and post 6-week intervention values and mean change (95% CI) in maximal oxygen uptake (absolute and relative values), and maximal aerobic speed after the intervention program. **(A,B)** Maximal oxygen uptake [VO_2_max (ml^∗^min^−1^)]; **(C,D)** maximal oxygen uptake [VO_2_max (ml^∗^kg^−1∗^min^−1^)]; **(E,F)** maximal aerobic speed [MAS (km^∗^h^−1^)]. ^§^ *P* < 0.05, ^§§^ *P* < 0.01, ^§§§^ *P* < 0.001 (analysis pre–post; Student’s paired *t*-test). ^∗^*P* < 0.05, ^∗∗^*P* < 0.01, ^∗∗∗^*P* < 0.001 (analysis between groups; ANCOVA).

Figure 2 shows changes (post–pre) on maximal performance-related parameters by group. Student’s paired *t*-test showed no statistical changes in absolute values of VO_2_max in WB-EMS and CG, but clinically relevant in WB-EMS (104.01 ± 41.22 ml/kg/min, *P* = 0.082) (Figure 2A). However, relative values of VO_2_max significantly increased in WB-EMS (2.79 ± 0.89 ml/kg/min, *P* = 0.001), while no significant changes were registered in CG (−0.36 ± 1.08 ml/kg/min, *P* = 0.821) (Figure 2C). We observed significant changes when comparing WB-EMS with CG (*P* < 0.001) only in relative terms (Figures 2B,D).

**FIGURE 3:**
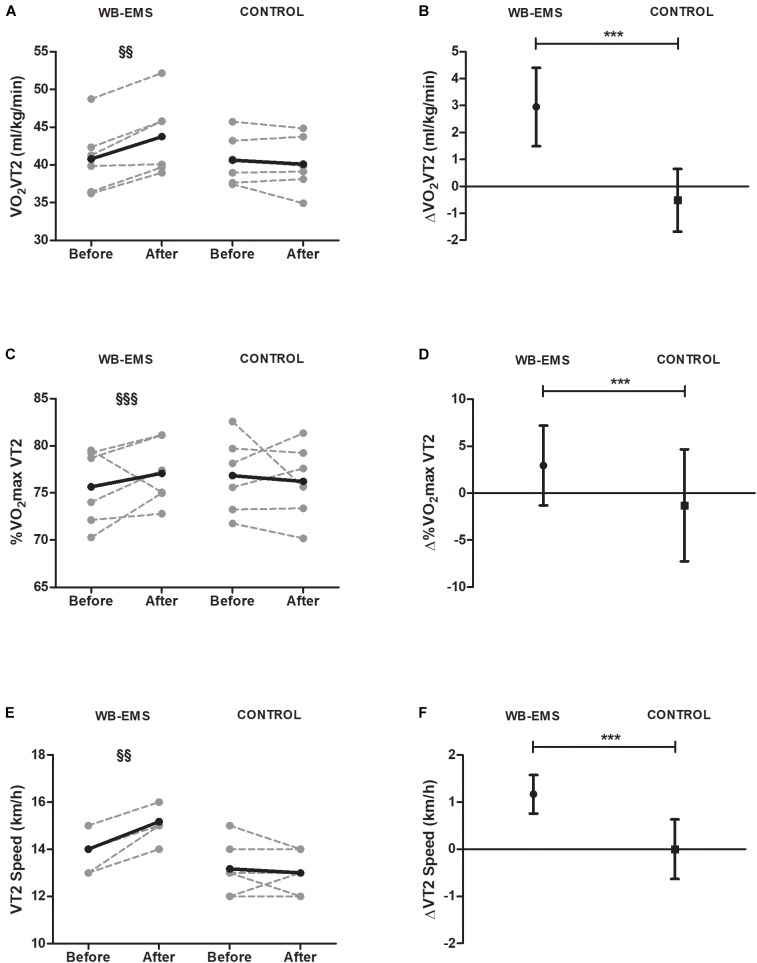
Pre and post 6-week intervention values and mean change (95% CI) in oxygen uptake at ventilatory threshold 2, maximal oxygen uptake percentage in ventilatory threshold 2, and ventilatory threshold 2 speed. **(A,B)** Oxygen uptake at ventilatory threshold 2 [VO_2_ VT2s (ml^∗^kg^−1∗^min^−1^)]; **(C,D)** maximal oxygen uptake percentage in ventilatory threshold 2 [%VO_2_max VT2]; **(E,F)** ventilatory threshold 2 speed [VT2 speed (km^∗^h^−1^)]. ^§^*P* < 0.05, ^§§^*P* < 0.01, ^§§§^*P* < 0.001 (analysis pre–post; Student’s paired *t*-test). ^∗^*P* < 0.05, ^∗∗^*P* < 0.01, ^∗∗∗^*P* < 0.001 (analysis between groups; ANCOVA).

ANCOVA revealed significant differences in maximal aerobic speed and VT2 speed when comparing changes of WB-EMS with CG (*P* < 0.05 for maximal aerobic speed and *P* < 0.001 for VT2 speed) (Figures 2E,F, 3E,F) but no differences were found in VT1 speed (*P* = 0.243).

Student’s paired *t*-test also showed significant increases in VO_2_ at VT2 in WB-EMS (*p* = 0.01) (Figure 3A). When compared mean change in WB-EMS with CG, we also observed significant differences (*P* < 0.001) (Figure 3B). Changes of the VO_2_max percentage reached in VT2 were also higher in WB-EMS when compared with CG (*P* < 0.001) (Figures 3C,D) and no changes were observed in VO_2_max percentage reached in VT1 (*P* = 0.517).

**FIGURE 4:**
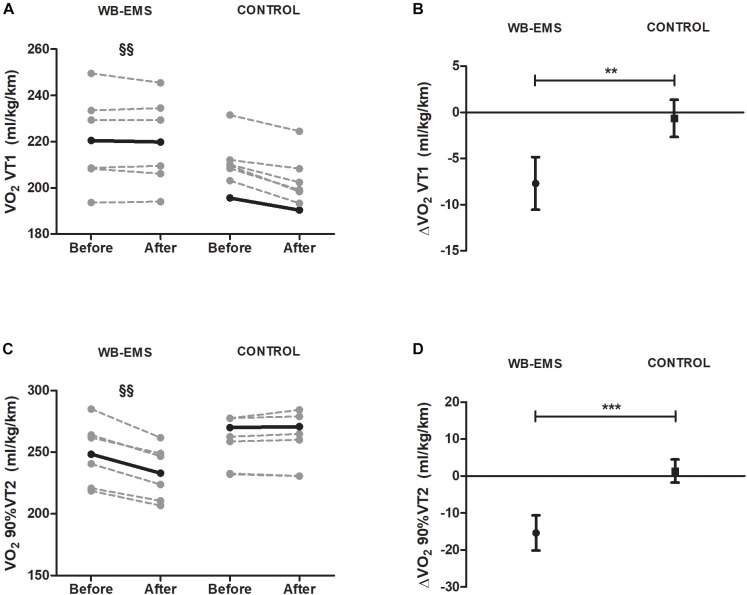
Pre and post 6-week intervention values and mean change (95% CI) in running economy at ventilatory threshold 1 and 90% of ventilatory threshold 2 speed after the intervention program. **(A,B)** Running economy at ventilatory threshold 1 speed [VO_2_ VT1s (ml/kg/km)]; **(C,D)** running economy at 90% of ventilatory threshold 2 speed [VO_2_ VT2s (ml/kg/km)]. ^§^*P* < 0.05, ^§§^*P* < 0.01, ^§§§^ *P* < 0.001 (analysis pre–post; Student’s paired *t*-test). ^∗^*P* < 0.05, ^∗∗^*P* < 0.01, ^∗∗∗^*P* < 0.001 (analysis between groups; ANCOVA).

Running economy at VT1 speed significantly increased in WB-EMS (−9.99 ± 3.80 ml/kg/min) compared with CG (*P* < 0.001; Figures 4A,B). Running economy at 90% of VT2 speed (Figures 4C,D) was also increased in WB-EMS (−15.38 ± 4.72 ml/kg/min, *P* = 0.01).

**FIGURE 5:**
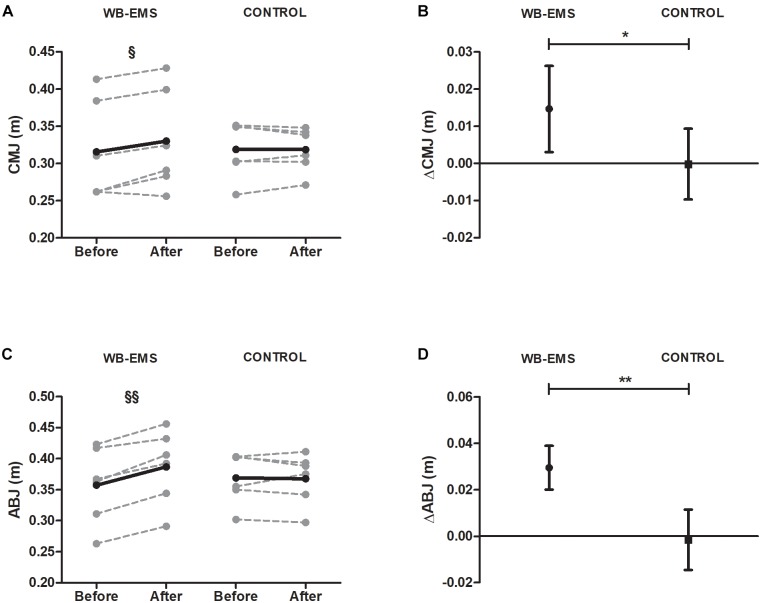
Pre and post 6-week intervention values and mean change (95% CI) countermovement jump and Abalakov jump after the intervention program. **(A,B)** Countermovement jump [CMJ (m)], **(C,D)** Abalakov jump [ABJ (m)]. ^§^ *P* < 0.05, ^§§^*P* < 0.01, ^§§§^*P* < 0.001 (analysis pre–post; Student’s paired *t*-test). ^∗^*P* < 0.05, ^∗∗^*P* < 0.01, ^∗∗∗^*P* < 0.001 (analysis between groups; ANCOVA).

Muscular power, assessed by vertical jump (CMJ and ABJ), improved in WB-EMS (0.02 ± 0.02 m in CMJ and 0.03 ± 0.01 m in ABJ, respectively), while no changes were observed in CG (Figures 5A,C). Significant differences were found in CMJ and in ABJ changes between WB-EMS and CG (*P* < 0.05 and *P* < 0.01, respectively) (Figures 5B,D).

## Discussion

The major findings of this study were that 6 weeks of WB-EMS training (coupled to a reduction in running endurance training) improved: (i) VO_2_max (5.2%); (ii) speed and VO_2_max percentage at which VT2 is reached (Δ = 8.6% and Δ = 4.6%, respectively); (iii) running economy at speeds where VT1 and 90% of VT2 were reached (−3.3% and −6.2%, respectively); and (iv) muscular power in CMJ and ABJ (Δ = 4.4% and Δ = 8.4%, respectively). These results suggest that, in recreational runners, WB-EMS training increased performance in spite of the significant reductions in endurance training, provided that WB-EMS training program follows a specific periodization of electrical parameters and is based on functional exercises.

### Maximal Oxygen Consumption

VO_2_max is not only an excellent marker of running performance (Bassett and Howley, 2000) but also a marker of health and quality of life (Kodama et al., 2009; McKinney et al., 2016). Available evidence suggests that aerobic endurance training, at an intensity of at least 65% of VO_2_max is an adequate stimulus to improve VO_2_max in individuals with baseline aerobic capacities below 40 ml/kg/min (Swain and Franklin, 2002). This finding was confirmed by a meta-analysis which revealed that the training effect of this modality was greater for less fit runners and with longer duration interventions (Milanović et al., 2015). On the other hand, it has been shown that other fitness training programs, such as HIIT, improved cardiovascular fitness and other fitness parameters (strength, body composition, or physical appearance) applying lower volume but higher intensity training. For this reason, HIIT is suggested to be a viable alternative to the traditional approach of continuous endurance training (Milanović et al., 2015). There are no plausible studies examining the effect of WB-EMS on cardiorespiratory fitness. However, Nuhr et al. (2003) found that the chronic application of local electromyostimulation (10 weeks, 4 h per day, 7 days per week) at low frequency (15 Hz) of the knee extensor and hamstring muscles of both legs in healthy volunteers improve maximal aerobic-oxidative capacity and VO_2_ at the anaerobic threshold 26% and 20%, respectively. In our study, we expected a decrease in VO_2_max as a result of the reduction applied in running training session; paradoxically an increase of 5.2% in relative values of VO_2_max was observed after 6 weeks of WB-EMS. This effect was similar to an intervention program consisting of 24 sessions of HIIT in people with cardiometabolic disorders (2–5 min at 80% VO_2_max with active recovery at 60% VO_2_max) and a bit smaller than the effects determined by 4 weeks of HIIT (4 × 4-min interval training at 90–95% HRmax with 3 min of active resting periods at 70% HRmax between each interval) in adults that elevated VO_2_max by 7.5% (Adamson et al., 2014).

### Ventilatory Thresholds

VT1 and VT2 speed performance is associated with muscle respiratory capacity, and the ability to produce less lactate at a given running speed is a determinant of prolonged running performance. It has been suggested that the submaximal blood lactate response to exercise is associated with peripheral factors including muscle fiber type, capillary density, and mitochondrial volume density (Esfarjani and Laursen, 2007). In the present study, speed at which VT1 and VT2 are reached were increased in WB-EMS (8.9% and 8.3%, respectively) compared with baseline values. These results are slightly smaller than those obtained in a specific HIIT intervention [high-intensity running bouts at 15.7 (0.7) km/h for 3.5 (0.7) min followed by low intensity recovery runs at 7.8 (0.3) km/h for 3.5 (0.7) min] in moderately trained young males (improvement of 11.7% in VT2 compared to baseline) (Esfarjani and Laursen, 2007). The increased mitochondrial enzyme content, which is associated with an increase in the rate of lipid utilization and consequently a decrease in rate of glycogen depletion and increased capillary density after endurance training, could also increase the exchange area and decrease the distance between the site of lactate production and the capillary wall, which could improve the lactate exchange ability (Esfarjani and Laursen, 2007). These are possible explanations for the increase of speed at which VT1 and VT2 is reached, after the application of WB-EMS training. Other possible reasons that could explain the increase of VT2 speed are the improvement of (i) running economy, (ii) VO_2_max, and (iii) maximal oxygen uptake percentage in VT2. Our results suggest that if we examined WB-EMS parameters, we could remark that improvements in VT2 speed could be the consequence of the sum of the three previous factors.

### Running Economy

Running economy refers to the oxygen uptake required at a given absolute exercise intensity (Barnes and Kilding, 2015). We have not found previous studies analysing the effects of WB-EMS in RE. However, it is well known that other training methodologies produce improvements in running economy. A traditional 14-week strength training protocol (six exercises, three to five sets, three to five reps, % 1RM) added to typical endurance training (concurrent training) produced a significant improvement in RE (5.6%) in middle-level young athletes (Guglielmo et al., 2009). In addition, explosive strength based on sprints (20–100 m) and plyometric training during 9 weeks also induced a significant improvement in RE (8.0%) comparing with a control group (no exercise) (Guglielmo et al., 2009). On the other hand, a HIIT protocol (3 min at 60–65% of maximal heart rate followed by four bouts alternating 4 min at 90–95% maximal heart rate and 3 min at 60–65% maximal heart rate, during 3 weeks) produced improvements in running economy at VT2 speed (8.8%) in healthy, physically active adults (Holmes et al., 2015). In our study, RE improved after the implementation of an intervention program with WB-EMS in two levels: VT1 speed and 90% of VT2 speed. The differences in both cases were a decrease of 3.3% VO_2_ in the VT1 speed and 6.2% in the 90% of VT2 speed intensity. Possible reasons for these results could be: (i) improvement of lower limb coordination and co-activation of muscles, increased leg stiffness and decreased stance phase contact times, allowing a faster transition from the braking to the propulsive phase through elastic recoil; (ii) changes in the nervous system increasing the activation capacity of the working muscles, thus producing a greater net force with each stride; (iii) increasing motor unit recruitment and motor unit synchronization that could improve mechanical efficiency and motor recruitment patterns; (iv) that greater muscular strength following strength and electromyostimulation training could produce a delay in muscular fatigue, resulting in a smaller increase in oxygen uptake (increased RE) at any given speed during sustained running (Foster and Lucia, 2007; Barnes and Kilding, 2015).

### Muscular Power

Muscular power is considered a critical element for carrying out daily activities and occupational tasks, as well as successful athletic performance and usually it is measured by vertical jump (Markovic, 2007). In our study, we analyzed the effects on two types of jump that allow differentiation between the elastic muscle component (CMJ) and coordinative jump component (ABJ). Both CMJ and ABJ improved after WB-EMS (4.4% and 8.4%, respectively). These results were similar to those showed in a meta-analysis, which concluded that plyometric training improved vertical jump performance, with CMJ improvements between 7 and 10.4% and ABJ improvements between 6.2 and 10.8% (Markovic, 2007). On the other hand, a 4-week local electromyostimulation in quadriceps combined with plyometric training program [16 sessions, four times per week: eight electromyostimulation training sessions (two each week) and eight plyometric training sessions (two each week)] increased CMJ (8.7%) (Herrero et al., 2006). The results of our study could be explained by neuromuscular adaptations, such as increased neural drive to the agonist muscles, improved intermuscular coordination, changes in the muscle–tendon mechanical-stiffness characteristics, changes in muscle size or architecture, and changes in single-fiber mechanics produced for the training protocol (de Villarreal et al., 2009). Of note, WB-EMS included a strength phase (thought to improve muscular power), HIIT which include plyometric exercises (thought to improve biomechanical parameters such us elastic muscular component), HIIT (thought to improve ventilatory and metabolic parameters), being all of them accompanied by WB-EMS (thought to improve neural activation of the trained muscles).

## Limitations

Our study has some limitations: (i) lack of food intake control; (ii) we cannot extrapolate the study results to well-trained athletes or sedentary population because our sample only includes male recreational runners; (iii) the absence of an additional group which includes WB-EMS while maintaining previous endurance training prevents us from knowing if these effects are equal or better when the WB-EMS is added to the habitual running training; (iv) we cannot measure health biomarkers which confirm the absence of high values of creatine kinase levels and/or rhabdomyolysis, yet participants did not report muscle pain or fatigue; (v) participants had no previous experience with WB-EMS training and we do not know whether these results extent to athletes with previous WB-EMS experience; (vi) the small sample size and therefore the low statistical power; (vii) the assignment of a WB-EMS effects in our study is difficult since we combined a variation of multiple stimuli (strength, power and HIIT training modalities); (viii) we did not use belts and cable to ensure speed actions like power-training. However, we strongly encouraged participants to perform each power action as fast as possible.

## Conclusion

In conclusion, our results suggest that a 6-week WB-EMS training program (six training sessions) combined with a significant reduction in endurance training, improved VO_2_max, VT1, VT2, RE, and vertical jump, which are related to running performance in recreational runners. Therefore, WB-EMS could be an effective training methodology to produce improvements in performance of recreational runners despite reductions in endurance training and to avoid detraining when aerobic training is reduced for certain reasons.

WB-EMS once per week combined with a relatively low volume of endurance training can be used to improve physiological performance attributes and muscular power capacities within a relatively short time period in male recreational runners. This study shows that a functional running structure of WB-EMS programming is able to improve VO_2_max, VT1, VT2, RE, and vertical jump over a 6-week period. For optimal adaptation and development of endurance and muscular power qualities, WB-EMS sessions should be carefully programmed considering the load, volume, and intensity of other training sessions without WB-EMS. In addition, it could be interesting to evaluate whether a combined WB-EMS and other training program produce extra improvements when controlling for confounders variables (physical activity, nutrition, rest, etc.). Inasmuch, when training cessation are superimposed (muscle discomfort, injuries, environmental conditions, etc.), WB-EMS could be a feasible and effective training alternative to prevents not only detraining consequences, but even to increase performance. We do not know whether these results can be extended to elite athletes, since the scope of performance in these individuals are lower. However, the fact that the application of WB-EMS is a novel stimulus could derive in an increment of running performance of the same magnitude as in recreationally runners. Moreover, further studies are needed to examine the physiological mechanisms that produce these improvements, and to well-understand if the improvement of endurance performance-related parameters observed in our study, are dependent of the exercises selected, the electrical parameters, or their combination.

## Data Availability Statement

The raw data supporting the conclusions of this manuscript will be made available by the authors, without undue reservation, to any qualified researcher.

## Author Contributions

FA-G, AD-l-O, LR-G, JR, and AG conceived and designed the study. FA-G, AD-l-O, LR-G, and AG designed the tests and did the intervention training. FA-G, GS-D, and JR elaborated the statistical section. FA-G drafted the manuscript. AG and JR revised the manuscript. All authors read and approved the final manuscript.

## Conflict of Interest Statement

The authors declare that the research was conducted in the absence of any commercial or financial relationships that could be construed as a potential conflict of interest.
